# Application of 20% silver nanoclusters in polymethacrylic acid on simulated dentin caries; its penetration depth and effect on surface hardness

**DOI:** 10.1038/s41598-023-48519-1

**Published:** 2023-11-30

**Authors:** Maria Belén Cabalén, Gustavo Fabian Molina, Vincent Piscitelli, Maximiliano Rossa, Juan Pablo Aranguren, Santiago Daniel Palma, Gustavo Ariel Pino, Mariana Picca, Michael Francis Burrow

**Affiliations:** 1 Becaria CONICET, Facultad de Ciencias de la Salud, Universidad Católica de Córdoba, Córdoba, Argentina; 2 Facultad de Ciencias de la Salud, Universidad Católica de Córdoba, Córdoba, Argentina; 3 The Faculty of Dentistry, University of Hong Kong, Pok Fu Lam, Hong Kong SAR China; 4 Centro Láser de Ciencias Moleculares, Universidad Nacional de Córdoba, Haya de La Torre S/N, Pabellón Argentina, Ciudad Universitaria, X5000HUA Córdoba, Argentina; 5 INFIQC: Instituto de Investigaciones en Fisicoquímica de Córdoba (CONICET – UNC), Universidad Nacional de Córdoba, Haya de la Torre S/N, Pabellón Argentina, Ciudad Universitaria, X5000HUA Córdoba, Argentina; 6 Departamento de Fisicoquímicas, Facultad de Ciencias Químicas, Universidad Nacional de Córdoba, Haya de la Torre S/N, Pabellón Argentina, Ciudad Universitaria, X5000HUA Córdoba, Argentina; 7 Cátedra de Materiales Dentales, Facultad de Odontología, Universidad de Buenos Aires, Buenos Aires, Argentina

**Keywords:** Dental caries, Biomineralization

## Abstract

The aims of this study were: To evaluate the surface hardness of simulated dentin caries lesions treated with either silver nanoclusters (AgNCls) synthesized in polymethacrylic acid (PMAA) or 38% silver diammine fluoride (SDF), as well as observe the penetration of the treatment solutions into the simulated caries lesions. Dentin blocks 4 mm thick obtained from caries-free third molars were sectioned and then simulated caries lesions on the occlusal dentin surfaces were created. Each specimen (n = 8) was divided into four sections: (A) treated with 20% AgNCls/PMAA; (B) treated with SDF 38% (FAgamin, Tedequim, Cordoba, Argentina); (C) sound tooth protected by nail-varnish during artificial caries generation (positive control); and (D) artificial caries lesion without surface treatment (negative control). AgNCls/PMAA or SDF were applied on the simulated lesions with a microbrush for 10 s, then excess removed. The surface hardness was measured by means of Vickers indentation test. To trace the depth of penetration, up to 400 μm, of silver ions, elemental composition of the samples was observed using EDX, coupled with SEM, and measured every 50 μm from the surface towards the pulp chamber. Laser Induced Breakdown Spectroscopy (LIBS) was also employed to trace silver ion penetration; the atomic silver line 328.06 nm was used with a 60 μm laser spot size to a depth of 240 μm. Student’s-*t* test identified significant differences between treatment groups for each depth and the Bonferroni test was used for statistical analysis of all groups (p < 0.05). Mean surface hardness values obtained were 111.2 MPa, 72.3 MPa, 103.3 MPa and 50.5 MPa for groups A, B, C and D respectively. There was a significant difference between groups A and C compared with groups B and D, the group treated with AgNCls/PMAA achieved the highest surface hardness, similar or higher than the sound dentin control. A constant presence of silver was observed throughout the depth of the sample for group A, while group B showed a peak concentration of silver at the surface with a significant drop beyond 50 μm. The 20% AgNCls/PMAA solution applied to simulated dentin caries lesions achieved the recovery of surface hardness equivalent to sound dentin with the penetration of silver ions throughout the depth of the lesion.

## Introduction

The increasing trend for preserving the structure of caries lesions using agents such as Silver Diammine Fluoride as part of the initial treatment has promoted the resurgence of non-surgical strategies for the recovery of enamel and dentin affected by the demineralization process. The most commonly used concentration of SDF is 38% (44,800 ppm F) with a reported clinical efficacy in arresting progression of dentinal caries found to be around 65.9%^1^. SDF in topical solutions has been shown to form silver phosphate that, when applied to carious lesions, rapidly turns black under the influence of reducing agents such as sunlight. The oxidation that occurs within the dental structures, which is the cause of the dark staining, is a controversial feature of SDF^2^. The discoloration effect on carious teeth is the most distinct deficiency of SDF and may therefore limit its clinical use in patients demanding more esthetic treatment outcomes.

Efforts have been made to counteract the staining effect of SDF by applying potassium iodide (KI) in a second step to prevent staining through the precipitation of excess silver ions as white silver iodide crystals^3,4^. Although most of the reports show that KI application after SDF treatment significantly reduced tooth staining for a short time, others suggest masking the SDF-treated surface with a tooth-colored restorative material, namely resin-modified or high viscosity glass ionomer cement, or a resin composite, with variable success as a darkened marginal stain typically remains if not tooth preparation occurs^3,5^. Potassium fluoride (KF) and silver nitrate (AgNO_3_) have also been proposed to mitigate SDF-mediated staining as well as polyethylene glycol (PEG)-coated nanoparticles containing sodium fluoride (NaF), and nanosilver fluoride (NSF)^5^.

The dentin architecture is disrupted when a caries lesion develops through the process of demineralization. Remineralization in dentin may take place when minerals interact with the undamaged organic matrix of the dentin. However, remineralization becomes more difficult when the matrix is more abundant. More than 90% of the organic component in dentin is Type I collagen, which provides the structural framework for apatite deposition and facilitates the enhancement of the mechanical properties of dentin^6,7^. Dentin collagen mineralization can be extrafibrillar and intrafibrillar. The former occurs in the intervals between collagen fibrils, while the latter occurs in the gaps, with the depositing minerals extending into the fibrils. In nature, only intrafibrillar mineralization in collagen fibrils may reproduce the original hierarchy of the mineral structure as in sound dentin, resulting in its optimized physical properties.

According to Minimally Invasive Dentistry principles, since the outer layer (the old term of infected dentin) is irreversibly denatured and cannot be remineralized, it should be removed; however, the inner layer (caries-affected dentin) can be remineralized^8^. Moreover, the remineralization of demineralized dentin is critical for improving bonding stability and preventing primary and recurrent caries lesions. On the other hand, laboratory research has indicated that only mineralized collagen fibrils can stop the degradation caused by MMPs and aging, and can thus restore the hardness of natural mineralized dentin, remove the action of these enzymes, and maintain the stability of the resin–dentin interface^9^. Functional mineralization by means of polymer induced liquid precursors (PILP)^10^ has been proposed to achieve intrafibrillar remineralization whereas the mechanism of SDF in arresting dental caries seems to be supported by extrafibrillar mineralization combined with the inhibition of the growth of cariogenic pathogens.

To avoid staining of the recovered structures, a prototype infiltrating/remineralizing solution was developed using silver nanostructures that do not undergo this oxidation. Preliminary studies showed that silver nanoclusters synthesized in polymethacrylic acid (AgNCls/PMAA) did not generate color changes in artificial caries lesions in dentin, compared to a solution of 38% silver diammine fluoride. Furthermore, the application of this solution significantly increased the adhesion of a glass ionomer restorative cement^11^. As mentioned, the vehicle of the silver nanoclusters is a polymeric acid, similar to those applied with the PILP prototypes to achieve functional (intrafibrillar) mineralization. Therefore, it was hypothesized that, in addition to the improvements in optical and adhesive properties, this solution of 20% AgNCls/PMAA may increase surface hardness of artificial caries lesions as well as evenly penetrate throughout the depth of the lesion.

In order to extend the assessment of other properties to characterize the development of this solution as a potential non-restorative treatment option for caries lesions, the effect of its topical application on a demineralized dentin surface as well as the penetration into the demineralized dentin were investigated. The aims of this study were: (a) to compare the surface hardness of artificial caries lesions in dentin treated with either 20% AgNCls/PMAA or 38% SDF, and (b) to evaluate the depth of penetration of silver ions into the demineralized dentin using either treatment option.

## Materials and methods

All experimental protocols were approved by the secretary of research and development, Universidad Católica de Cordoba, Argentina (SI-UCC research grants) and by the National Agency for Research under the research grant FONCYT-PICT2020 Serie A #00539, and PICT2019 N 241, CONICET-PIP, PRIMAR2017 (SECyT/UNC). All methods were carried out in accordance with relevant guidelines and regulations.

### Preparation of samples

Two batches of eight non-carious third molars were obtained from the Bank of Human Teeth, Faculty of Dentistry, Universidad Nacional de Cordoba, Argentina (Ord.3/16 HCD and Res. 333/17 HCD); one batch was used for the Microhardness test whereas the second batch, for tracing silver ions using LIBS first, followed by EDX analysis.

Dentin blocks, 4 mm thick, were obtained by removing the occlusal enamel using a water-cooled low-speed cutting machine (Buehler, Germany) perpendicular to the long axis of the tooth to obtain flat mid-coronal dentin surfaces. These were sequentially polished with 400 to 1200-grit silicon carbide papers followed by coating with nail varnish (Revlon, New York, USA) to leave an exposed window 5 × 5 mm on the occlusal dentin surface for production of demineralized dentin to simulate dental caries and preserving the sound dentin surfaces covered by the nail varnish for later comparison.

Samples were immersed for 66 h in a solution containing 0.05 M acetate buffer, 2.2 mM calcium phosphate adjusted to pH 5.0 to generate a demineralized layer of approximately 150–200 μm deep to simulate a carious lesion^12^.

**Figure 1:**
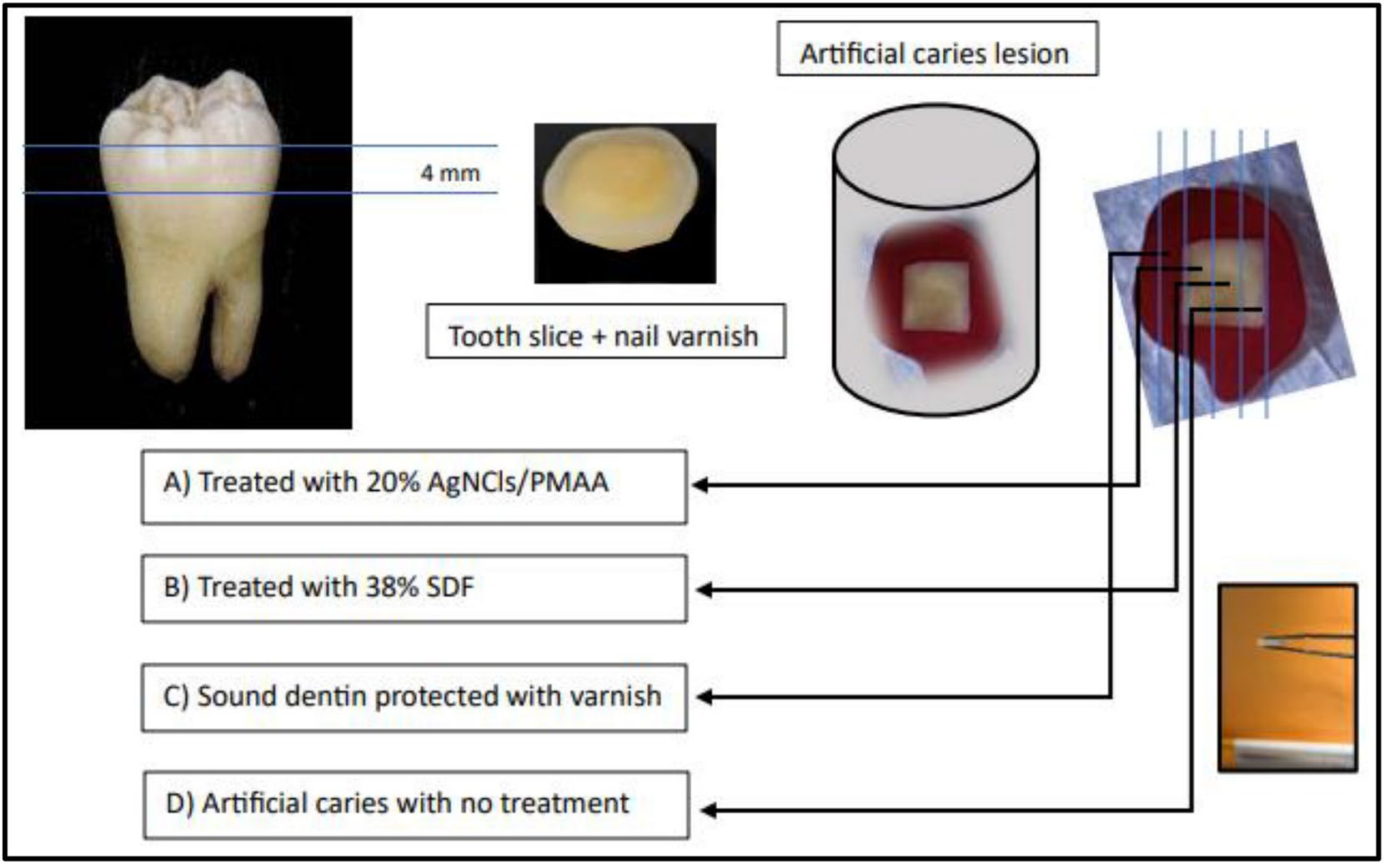
Preparation of the samples.

Once the artificial lesions were produced, four slices 1.5 mm thick from each specimen were cut from occlusal to apical region, and carefully secured in a positioner shelf to receive either treatment as displayed in Fig. 1. Specimens were divided into the following groups:

1. Treated with 20% AgNCls/PMAA^11^; the solution was applied to the exposed demineralized surface with a microbrush for 10 s then incubated at 37 °C and 100% relative humidity for 24 h;
2. Treated with SDF 38% (FAgamin, Tedequim, Cordoba, Argentina); the solution was applied on the exposed demineralized surface with a microbrush for 10 s, left to rest for 180 s and excess was removed with a cotton pellet^13^, then incubated at 37 °C and 100% relative humidity for 24 h;
3. Sound tooth structure protected by nail varnish during artificial caries generation and incubated for 24 h at 37 °C and 100% relative humidity (positive control); and,
4. Control (no treatment); exposed demineralized surfaces were left untreated then incubated for 24 h at 37 °C and 100% relative humidity (negative control).

### Testing the samples

#### Surface hardness

The hardness test was performed at room temperature by the Vickers indentation test, using a hardness tester (Microhardness Tester FM-300; Future-Tech Corp, Fujisaki, Kanagawa, Japan) consisting of a pyramidal diamond indenter.

Vickers hardness (HV) values were determined by performing 5 indentations in different locations on each specimen with a load of 100 g for 10 s on the polished surface.

#### Depth of penetration of silver ions

##### Laser Induced Breakdown Spectroscopy

**Figure 2:**
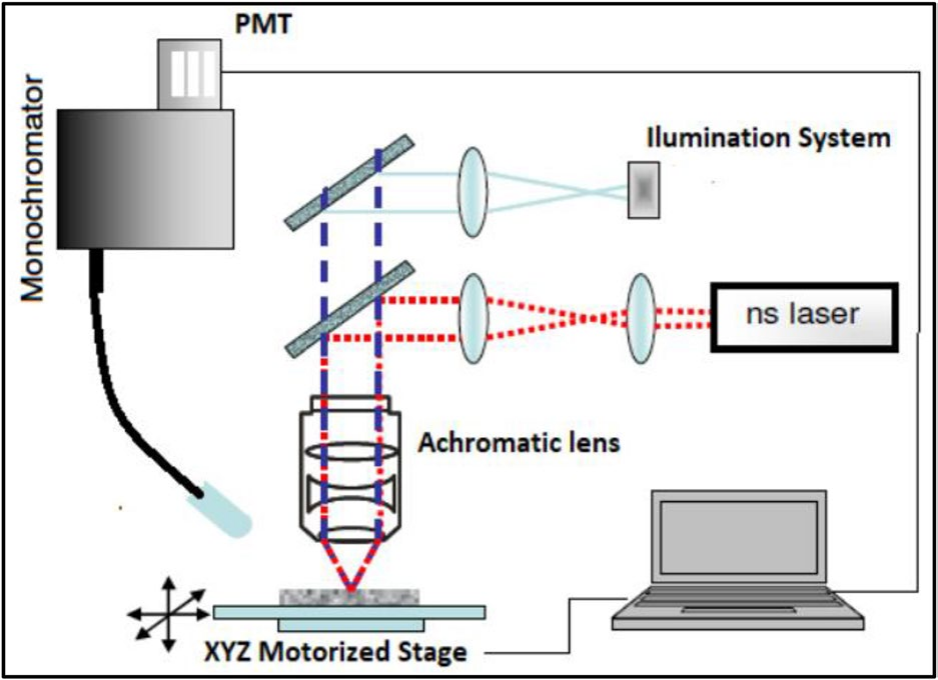
Experimental setup for LIBS.

A diagram for the Laser Induced Breakdown Spectroscopy (LIBS) system is shown in Fig. 2. A Q-switched Nd:YAG laser (Surelite I, Continuun), operating at its third harmonic wavelength (355 nm) was used to initiate the ablation. The laser beam was directed to the sample using dichroic mirrors and was focused using a 5 cm focal length achromatic lens. The sample was positioned at the focal plane of the lens in order to create craters close to the optical diffraction limit. An XYZ motorized stage was used for positioning the sample. Images of the sample were acquired by an instrument coupled webcam. A UV–Vis Fiber optic and collimator were used to image the laser-induced plasma into the entrance slit of a Czerny-Turner monochromator (McPherson model 218, 0.3 m) equipped with a 1200 groove/mm grating. Spectral emission was detected by a PMT (Hamamatsu R636-10).

A spot size of 60 μm was used and 8 sampling spots separated 30 μm apart from each other were studied to achieve a penetration depth of 240 μm. A silver atomic line at 328.06 nm was selected since spectral interference was not observed and it is one of the most intense emission lines for silver.

##### SEM/EDX

SEM analyses were subsequently performed as an alternative procedure to measure the depth of penetration of silver ions using the same specimens. Images were acquired using a Philips SEM (XL30, Netherlands). The elemental composition of the samples was observed using EDX, coupled with SEM. Specimens were sputter-coated with gold (Q150R ES, Quorum Technologies, East Sussex, UK) with an operating current of 23 mA. The specimen surface was then examined using a scanning electron microscope (SEM, JSM 7800F, JEOL Ltd., Tokyo, Japan) with an accelerating voltage of 5 kV and a magnification ranging from 2500 × to 20,000 ×.

Elemental analysis was performed using a dispersive X-ray spectrometer (EDX, X-Max 20, Oxford Instruments, Abingdon, UK) to determine the elemental composition (Ca, P, Ag, or F) of the precipitate on representative specimens. The chemical element mapping was performed using EDX, tracing the silver signal. Three random lines from the outer surface of the lesion and up to 400 μm deep served to quantify the presence of silver at 50 μm intervals at a magnification of 20,000 × and a beam voltage set at 5 kV. An average elemental value was expressed as %weight/volume of silver, and recorded for each depth. A quantitative analysis of the presence of silver ions was performed at 50 μm increments up to 400 μm from the surface (8 sub-segments).

### Statistical analysis

For the Microhardness test, the average of the five measurements obtained on each specimen was recorded in an Excel file. Statistical analysis was performed using ANOVA with the significance set at the 95% confidence level (p < 0.05). Statistical differences between groups for surface hardness were determined using the Bonferroni test (p < 0.05). To determine differences between the two treatment options at each depth, Student’s-*t* test was used with significance set at p < 0.05, whereas significant concentration differences at all depths of penetration were determined by means of the SNK test set at α = 0.05.

### Statement of ethics

All experimental protocols were approved by the secretary of research and development, Universidad Católica de Córdoba, Argentina (SI-UCC research grants) and by the National Agency for Research under the research grant FONCYT-PICT2020 Serie A #00539, and PICT2019 N 241, CONICET-PIP, PRIMAR2017 (SeCyT/UNC). All methods were carried out in accordance with relevant guidelines and regulations.

Informed consent was obtained from all subjects and/or their legal guardian(s) who donated their teeth to the Bank of Human Teeth, Faculty of Dentistry, Universidad Nacional de Cordoba, Argentina (Ord.3/16 HCD and Res. 333/17 HCD).

## Results

### Surface hardness

Table 1 shows the mean values obtained for each experimental group A, B, C and D being, 111.2 MPa, 72.3 MPa, 103.3 MPa and 50.5 MPa respectively. The results showed significant differences between groups A (treated with AgNCls/PMAA) and C (sound dentin) in relation to groups B (treated with SDF) and D (demineralized dentin) (p = 0.01). The group treated with the 20% AgNCls/PMAA solution achieved the highest microhardness value, similar to/or higher than that of sound dentine.

**Table 1:** Surface hardness mean values and standard deviations of the four groups.

Treatment group	N	VH (MPa)	SD
A: AgNCls/PMAA	8	111.2^c^	8.2
B: SDF	8	72.3^b^	6.4
C: Sound dentin	8	103.3^c^	7.2
D: Artificial lesion	8	50.5^a^	3.0

Values expressed in megapascal (MPa); Different letters express statistical differences p < 0.05.

### Depth of penetration of silver ions

EDX was used to verify the presence of elemental silver in the SEM images of groups C and D. The EDX analyses confirmed the lack of detectable silver in non-demineralized samples as well as in untreated control lesions. While for groups A and B, the depth of penetration from 0 to 400 µm of silver quantified by weight % was verified.

**Figure 3:**
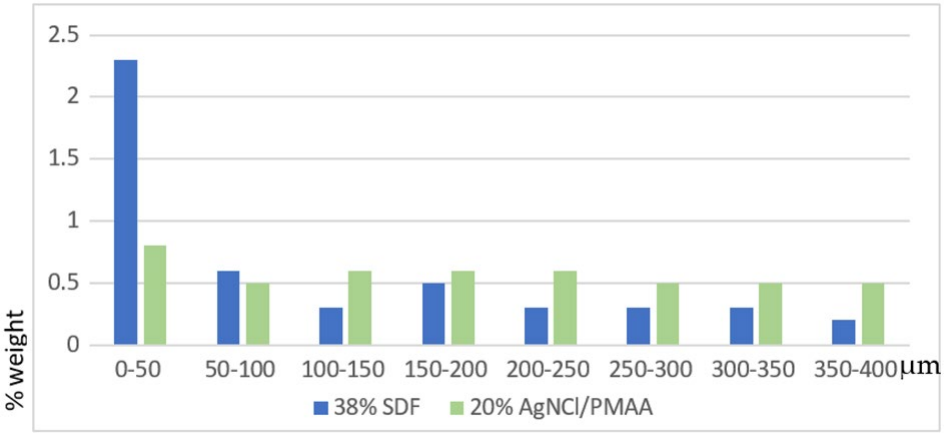
Penetration of silver ions into different depths of the treated dentin measured with EDX.

As shown in Table 2 and in Fig. 3, there is a greater presence of silver on the surface ranging from 0 to 50 µm for the group treated with 38% SDF, while the group treated with 20% AgNCl/PMAA maintained significant and constant amounts of silver throughout the entire lesion depths, from 0 to 400 µm.

**Table 2:** Mean (and standard deviation) %weight per volume values of silver traced at different penetration depths for AgNCls/PMAA and SDF groups.

Depth	AgNCls/PMAA		SDF		*p* value
	Mean	SD	Mean	SD	(Student-t)
0–50 µm	0.8^c^	0.16	2.3^d^	0.09	< 0.0001*
50–100 µm	0.5^b^	0.07	0.6^b^	0.03	0.67
100–150 µm	0.6^b^	0.07	0.3^a^	0.1	< 0.0001*
150–200 µm	0.6^b^	0.15	0.5^b^	0.07	0.12
200–250 µm	0.6^b^	0.07	0.3^a^	0.16	0.0007*
250–300 µm	0.5^b^	0.09	0.3^a^	0.12	0.0025*
300–350 µm	0.5^b^	0.07	0.3^a^	0.11	0.001*
350–400 µm	0.5^b^	0.08	0.2^a^	0.05	< 0.0001*

Ref. Values expressed in %weight per volume. Different letters express significant differences between groups. For each depth of penetration, significant differences between AgNCls/PMAA and SDF groups were determined by means of Student-t test and p-value indicated in the respective row (*p < 0.05).

**Figure 4:**
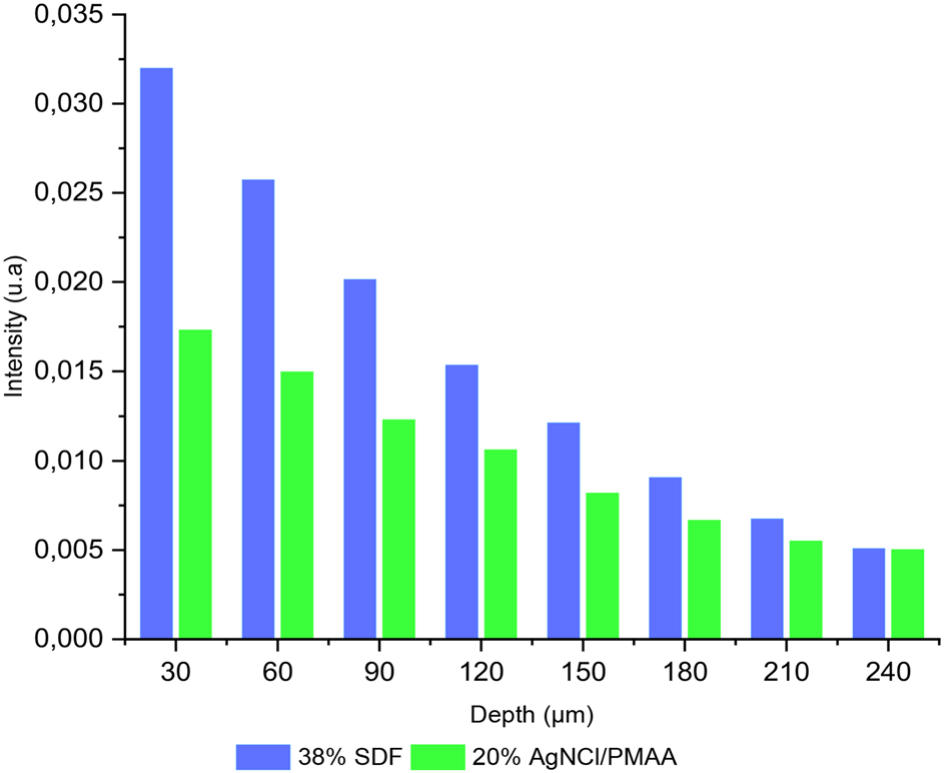
Intensity (328.06 nm silver atomic line) vs Penetration depths of the treated dentin measured with LIBS.

The analysis by LIBS was carried out on 8 points from one specimen from each treatment group, namely one specimen treated with 38% SDF and the other treated with 20% AgNCl/PMAA. For each sample 8 craters of one-pulse were made and the emission intensity of 328.06 nm line from Ag was recorded. The variation of the intensity of the silver atomic line by depth is expressed in Table 3 and Fig. 4.

**Table 3:** Mean (and standard deviation) Intensity atomic line 328.06 nm of silver traced at different penetration depths for AgNCls/PMAA and SDF groups.

Depth	AgNCls/PMAA		SDF	
	Mean	SD	Mean	SD
0–60	0.017	0.001	0.03	0.01
30–90	0.015	0.002	0.025	0.004
60–120	0.012	0.002	0.02	0.003
90–150	0.011	0.001	0.015	0.003
120–180	0.008	0.001	0.012	0.005
150–210	0.007	0.002	0.009	0.002

No statistical differences p < 0.05 were detected among these groups.

The intensity of the atomic line revealed a greater concentration of silver on the surface for each group. Although the group treated with 38% SDF initially showed higher intensity values compared with the samples from the 20% AgNCl/PMAA group, according to the reason that the silver concentration of the SDF solution is higher than the corresponding concentration of the AgNCl/PMAA solution. The readings of the SEM graphs show that, as depth increased, the silver concentration of the samples treated with 38% SDF decreased more rapidly than the samples treated with 20% AgNCl/PMAA. The silver concentration of the group treated with 20% AgNCl/PMAA remained almost constant and from 150 µm onwards the concentrations for both treatment groups were not statistically different (p > 0.05).

## Discussion/conclusion

It is suggested that, to recover functional and physical properties of dentin that can be remineralized, the remineralizing agent must reach the base of the lesion and deliver the chemical compounds from the bottom-up that will provide structural form and strength to the collagen network. However, how to measure the depth of penetration offers a challenge, even in laboratory models. Studies based on transverse microradiography alone provide information only on mineral density and do not provide ultrastructural evidence of intrafibrillar remineralization that is critical for restoring the mechanical properties of the remineralized dentin matrix. Thus, to test the hypothesis of this study, it used a combination of mineral density assessment by means of a scanning electron microscope (SEM) coupled with a dispersive X-ray spectrometer, and the evaluation of dynamic mechanical properties of the remineralized dentin by testing the microhardness of the surface exposed to the two remineralizing solutions.

The results obtained in the present study show similar values to other published data that found the Vickers hardness of natural dentin (ND) was 75.1 ± 3.2 MPa, decreasing to 55.2 ± 2.9 MPa after demineralization of the dentin (DD)^8^. In that study, when these surfaces were treated with a biomimetic mineralizing solution, the demineralized dentin was remineralized, reaching hardness values of 68.5 ± 2.5 MPa in the biomimetic mineralized dentin (BMD) group. The BMD group also showed higher hardness values than the DD, the ND and conventional mineralized dentin.

In the present study, the highest values for surface hardness were observed in the AgNCls/PMAA group, which contains no fluoride in its composition. It is hypothesized that it was the PMAA that could have modified the disposition of collagen fibers, consolidating a tight and dense network with improved mechanical properties. In the evaluation of the hypothesis, one must also consider the limitations of the artificial caries model that has been universally employed for the evaluation of dentin remineralization. Unlike artificial carious lesions, naturally occurring caries-affected dentin produced by bacterial acid challenge in the oral cavity is highly heterogeneous and the dentinal tubules are occluded by minerals that restrict diffusion of large molecular species into the intertubular collagen matrix^14,15^.

Remineralization of demineralized dentin is attributed to the high concentration of fluoride in SDF whereas silver is responsible for the antibacterial effect. Silver ion penetration studies showed that it is distributed throughout the affected area, even penetrating into parts of the healthy dentin. This fact had already been demonstrated in previous work using SDF via an EDX technique^16^. The results of the current study show that both compounds penetrated the demineralized tissues, but the 38% SDF showed a less homogeneous distribution of silver ions than the samples treated with 20% AgNCl/PMAA. It is known that silver can inhibit bacterial growth as it interacts with bacterial cell membranes and enzymes as well as being able to dope the hydroxyapatite. This silver-doped hydroxyapatite increases the antibacterial effect and prolongs its presence over time^17^. As the samples treated with 20% AgNCl/PMAA showed a better and more homogeneous distribution of ions, it might be assumed that the antibacterial effect is likely to homogeneously cover the affected area.

The LIBS technique has been previously used in dental research to determine the presence or absence of carious or demineralized areas^18,19^ and to study the presence and distribution of elements in teeth^20,21^. In this study, LIBS proved its usefulness to determine the silver signals at different depths of penetration of the treated samples. These results correspond with those obtained from the EDX analyses. The differences observed from both techniques may be attributed to the fact that EDX determines %weight per volume while LIBS measures relative molar concentrations per volume. In addition, EDX analysis is more superficial than LIBS analysis, which means that in the latter case there is also a component of the lateral diffusion of SDF or AgNCl/PMAA. However, at variance with EDX, LIBS is a relatively simple and cheaper technique that can be set-up in any research laboratory.

Regular strategies for remineralization have been based upon the use of fluoride compounds to produce hypermineralization of the lesion surface. Top-down remineralization strategies invariably require the presence of non-collagenous proteins such as phosphophoryn and dentin matrix protein that are present during the formation of dentin. Partial demineralization of mineralized collagen fibrils by bacterial acids represents a top-down approach in generating apatite seed crystallites, which differs to intrafibrillar mineralization using polymer-induced precursors. Expressed in conventional crystallization terminology, current remineralization strategies lack the mechanisms for inducing apatite nucleation and hierarchical assembly of apatites within a collagen matrix^22^.

Guided tissue remineralization (GTR) represents a promising strategy in collagen biomineralization. This mineralization strategy, that is particle-mediated and that progresses from the bottom-up, is somewhat different to what has been traditionally used in dentistry for conventional remineralization techniques. This strategy utilizes nanotechnology and biomimetic principles to achieve intra- and extrafibrillar remineralization of a collagen matrix in the absence of apatite seed crystallites^14^. This may explain why the amount of silver decreased at a depth of 100–150 μm and increased again at 150–200 μm, instead of decreasing continuously, as shown in Fig. 3. As this increase can also be observed in the penetration of silver from the SDF, it may be hypothesized that these artificial lesions presented a more affected area at a depth of 150–200 μm, where silver particles were more easily allocated.

In the attempt to achieve functional mineralization, polyanionic analogs are involved to template the functions of dentin matrix proteins in biomineralization. For that purpose, a polycarboxylic acid-based biomimetic analog is usually employed as a sequestration agent to stabilize amorphous calcium phosphate. In the present study, polymethacrylic acid functions as the polymer liquid precursor and AgNCls plays the role of calcium and phosphate ions to reproduce this particle-based assembly approach in the absence of apatite seed crystallites in the collagen matrix.

This biomimetic remineralization process represents a bottom-up approach to create nanocrystals that are small enough to fit into the gap zones between adjacent collagen molecules, and to establish a hierarchical order within the mineralized collagen. To determine whether this type of remineralization occurs using this prototype solution will require more accurate techniques and resources, which is encouraged by the results obtained in this preliminary study.

Therefore, considering the above mentioned limitations, it is possible to conclude that the use of a 20% AgNCls/PMAA solution to treat artificial caries lesions was able to restore the hardness of demineralized dentin to the hardness of undemineralized dentin and was able to penetrate to the full depth of the demineralized lesion.

## Data Availability

The data that support the findings of this study are available from FONCYT-PICT Serie A 2020 but restrictions apply to the availability of these data, which were used under license for the current study, and so are not publicly available. Data are however available from the authors upon reasonable request and with permission of FONCYT-PICT Serie A 2020 and CONICET. The corresponding author should be contacted if someone wants to request the data from this study.
